# Methods for Quantifying and Characterizing Errors in Pixel-Based 3D Rendering

**DOI:** 10.6028/jres.113.017

**Published:** 2008-08-01

**Authors:** John G Hagedorn, Judith E Terrill, Adele P Peskin, James J Filliben

**Affiliations:** National Institute of Standards and Technology, Gaithersburg, MD 20899-8911

**Keywords:** computer graphics, metrology, pixel measurement, rendering measurement, scientific visualization, virtual measurement

## Abstract

We present methods for measuring errors in the rendering of three-dimensional points, line segments, and polygons in pixel-based computer graphics systems. We present error metrics for each of these three cases. These methods are applied to rendering with OpenGL on two common hardware platforms under several rendering conditions. Results are presented and differences in measured errors are analyzed and characterized. We discuss possible extensions of this error analysis approach to other aspects of the process of generating visual representations of synthetic scenes.

## 1. Introduction

The visual display of virtual three-dimensional (3D) scenes has become common-place in modern computing systems. Applications can be found in such diverse fields as consumer games, video production, advertising, computer-aided design, and scientific data visualization.

While many of these applications are intended to provide a qualitative experience to the viewer, others are intended to convey accurate representations of spatial relationships. Applications such as computer-aided design and scientific data visualization have important quantitative components. For example, at NIST, we have implemented interactive measurement tools into the virtual world [1]. As we use such systems for quantitative tasks, it is incumbent upon us to understand how the computer graphics environment contributes to uncertainty in these tasks. The work we present here is a first step toward this understanding.

In current computer graphics systems, virtual scenes are usually described internally as a set of geometric objects in a 3D coordinate system. These geometric descriptions are then transformed into a set of pixels that are displayed to the user on a monitor. The process of transforming the 3D descriptions to a set of pixels is referred to as *rendering*.

We have developed methods for assessing geometric errors introduced by the rendering process. While this is hardly the only possible source of error that may be introduced by computer graphics technology, in this paper we focus exclusively on the rendering process. We will not examine other possible sources of error such as inaccuracies in display devices or errors introduced by variations in user viewing conditions.

It is also worth noting that issues of inaccuracies in visual presentation are not confined to the display of computer generated pictures. The display of different types of content (such as photographs, motion pictures, 3D synthetic scenes, and conventional two-dimension al graphs) and different media (such as paper, film, cathode ray tube (CRT), flat panel, and video projection) could each have its own set of uncertainty issues. For example, CRT displays can exhibit pincushion and barrel distortions as well as other geometric errors [2].

## 2. Approach

We study the rendering of three 3D geometric primitive forms: points, line segments, and triangles. These represent the simplest and by far the most commonly used geometric forms rendered in current computer graphics systems. Our work starts from the assumption that for each of these 3D geometric forms, there is a correct projection of the form to a two-dimensional (2D) rectangle that represents the viewing area. The viewing area can be thought of as an idealization of (a portion of) the monitor screen, however it is not discretized into a finite set of elements (pixels) as a real screen is. This projection is described in many standard references on 3D computer graphics [3]. We use this projection from 3D coordinates to the 2D coordinates of the viewing rectangle as an idealized form of the rendering process. We measure rendering errors by comparing the set of pixels drawn by the rendering process with the projection of the primitive to the idealized viewing rectangle.

The rendering process is treated here as a *black box*. We examine the results of the rendering of points, line segments, and triangles without trying to evaluate the correctness of implementation of any specific rendering algorithm. We do not attempt to explain any particular behavior of the rendering system; we simply observe and measure it.

Furthermore, we consider only the positional accuracy of the rendering. In other words, we are looking only at the positions of the pixels that are drawn as part of each primitive. The accuracy of the color or intensity of the pixels is not considered.

## 3. Method

We perform three sets of experiments, one set for each of the three types of geometric primitives. Each experiment is a run of a program that renders a set of geometric primitives and compares the actual pixels rendered with the ideal projection of the primitive to the viewing area. For each primitive type, we compute error metrics that quantify the amount by which the rendering of a primitive deviates from its ideal projection. The error metrics used for each primitive type are described below.

We ran each experiment on two platforms. Both platforms use commodity hardware and operating systems. We will refer to these as *Platform I* and *Platform II*. We keep the platforms anonymous because we wish this paper to be a presentation of our methods rather than an evaluation of specific hardware and software.

In the experiments that we describe here, the programs render the primitives using OpenGL^1^ [4], however our method is not dependent on any particular rendering software or rendering technique. Indeed, these methods could just as easily be used with other 3D rendering libraries such as DirectX [5]. We chose OpenGL because it is readily available on many hardware platforms, and is used in many consumer, industrial, and research applications. Our test software was designed so that it could easily be ported to a variety of hardware platforms and operating systems. We have placed our software on the web [6].

Again, we want to emphasize that the purpose of this paper is not to determine whether one hardware or software platform is better than another, or to evaluate the performance of specific systems. Nor are we testing the correctness of the implementation of any rendering algorithm. Rather our intent is to describe methods by which one can characterize and quantify the errors associated with a given 3D rendering system and compare one system with another. In this paper, we use specific hardware and software as vehicles for testing these methods and for showing their efficacy.

The basic procedure for all of our experiments was:

- Randomly generate 8 sets of viewing parameters
- Randomly generate a set of geometric primitives
- For each primitive, each view, and each platform
  – render the element as white pixels on a black background without antialiasing
  – look at which pixels are part of the rendered element (using the OpenGL procedure glReadPixels)
  – quantify the deviation of the rendered element from the projection to the idealized viewing rectangle
- Aggregate errors for individual primitives to form statistics
- Compare error statistics across viewing parameter sets and across platforms

We use a portable random number generator [7] for creating the randomly distributed viewing parameters and geometric primitives. This ensures that we are running the same tests across platforms.

### 3.1 Generation of Viewing Parameters

Viewing parameters for 3D rendering can be expressed in terms of a camera metaphor. The camera has a location, an orientation and an angular field of view (FOV). The location is simply a point in 3-space. The orientation determines the direction the camera is pointing and which way is “up”. The angular field of view corresponds to the “length” of the virtual camera lens. For example, an angular field of view of 10 degrees would be like a telephoto lens (a “long” lens) while an angular field of view of 100 degrees would be like a wide-angle or fish-eye lens (a “short” lens). The field of view of this virtual camera forms an infinite four-sided pyramid in the 3D coordinate system. The four faces of this 3D pyramid correspond to the four edges of the 2D viewing area. Only geometry that intersects this pyramid can be rendered.

In addition to these camera-like parameters, there are several other parameters that describe viewing in a typical pixel-based computer graphics system. The size of the rectangular screen area that is used for rendering is described by its height and width in pixels. We will call this rectangular screen area the rendering window. Following the camera analogy, the 2D viewing area and the corresponding rendering window on the screen can be thought of as the focal plane of the virtual camera.

Also, many computer graphics systems require the definition of near and far clipping planes. These are planes in the 3D coordinate system that are typically perpendicular to the direction that the camera is pointing. They are usually specified by the distance to the plane from the view point (the camera location) along the view direction vector. Normally any geometry that is closer than the near clipping plane or beyond the far clipping plane is discarded (clipped). So the infinite four-sided pyramid described above is reduced to a truncated pyramid with six faces (a frustum).

For the tests described here, there are eight different combinations of viewing parameters: two camera orientations, two angular fields of view, and two window sizes. The two camera orientations are randomly generated; we refer to these as *CO-1* and *CO-2*. The two angular fields of view are 30 degrees and 150 degrees, and the two pixel dimensions are 128×128 pixels and 907×907 pixels. The camera location is fixed at the origin. The near and far clipping planes are set at 0.01 units and 10.0 units respectively away from the view point. We number these eight sets of viewing parameters sequentially 1 through 8 as indicated in the Table 1.

### 3.2 Coordinate Systems

There are three coordinate systems that we will use. The first coordinate system is the 3D coordinate system in which the geometric primitives (points, line segments, and triangles) are defined. Then there is the 2D *pixel coordinate system* of the rendering window. In this coordinate system, one unit of length is equal to the spacing between adjacent pixels in the X or Y direction. Note that this implies that the X spacing of pixels is the same as the Y spacing of pixels. Our methods as implemented assume this equality, although they are easily adapted to unequal pixel spacing in X and Y. We also use the pixel coordinate system for the 2D viewing area that represents an idealized form of the rendering window. Although this viewing area is not discretized into pixels, it is convenient to use the same pixel-based coordinate system.

In addition to the pixel coordinate system, we also introduce a 2D coordinate system for the rendering window that normalizes distances based on the size of the window. We define one unit of length in *normalized window coordinates* to be $\sqrt{HW}$ where *H* is the height of the window in pixels and *W* is the width of the window in pixels. This coordinate system enables us to compare distances (and areas) in windows of different sizes. Measurements expressed in either of these coordinate systems are easily converted to physical coordinates (such as millimeters) if the physical spacing between pixels is known.

As mentioned above, the projection from the 3D coordinate system to the 2D pixel coordinate system is a major part of the rendering process and is a central component of our methods. The normalized window coordinate system is used for defining normalized versions of our error metrics.

### 3.3 Generation of Geometric Primitives

All of the geometric primitives are specified in terms of points in the 3D coordinate system. A triangle is specified by its three 3D vertex points; a line segment is specified by its two 3D end points; and a point primitive, of course, is specified by one 3D point. For each primitive, we get the required number of points by generating points that are randomly distributed within the 3D unit sphere (a sphere of radius 1, centered at the origin). Each point is produced by randomly generating X, Y, and Z coordinates in the interval [−1, 1] and discarding those points that lie outside the unit sphere. This random distribution of points through a spherical volume (rather than, for example, through a cubical volume) ensures that the directions of the points relative to the center of the sphere are randomly distributed. The center of the sphere (the origin) is, as described above, our camera location for all of the eight sets of viewing parameters. For the tests described below, 100,000 of each geometric primitive are generated.

### 3.4 Error Metrics for Point Primitives

The 3D point primitive is regarded as a 0-dimensional object described by a set of X, Y, and Z coordinates. The rendered pixel for a given point primitive is regarded as a zero-dimensional object (a point) in the 2D pixel coordinate system of the screen.

We present the error metric for the rendering of a point primitive in both a non-normalized and a normalized form. The non-normalized metric is a length in pixel coordinates, the normalized form is a length in normalized window coordinates. The non-normalized metric is simply the distance from the projection of 3D point to the ideal viewing rectangle from the center of the rendered pixel in pixel coordinates. As mentioned above, we determine the coordinates of rendered pixels by using the OpenGL procedure glReadPixels, which enables us to examine the contents of pixels in the frame buffer after we render the primitive. This method is used for all of the metrics described below.

To specify the point rendering metrics, we begin with some definitions. Let

*p* be a 3D point,

*proj*(*p*) be the projection of point p to 2D pixel space,

*r*(*p*) be the 2D coordinates of the pixel rendered for 3D point p, and

*dist*2*D*(*p*1, *p*2) be the distance between 2D points p1 and p2.

We now can define the non-normalized point rendering error metric (*PE*) for a 3D point p:

PE(p)=dist2D(proj(p),r(p)). (1)

**Fig. 1:**
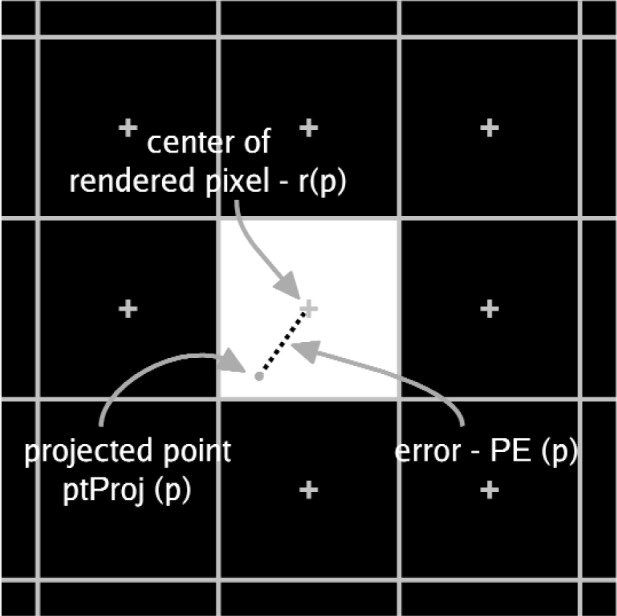
The point rendering error is the distance from the projection of the 3D point to the center of the rendered pixel.

Figure 1 illustrates the point error metric. This measure is in units of linear pixel coordinates.

We then take this point rendering metric and scale it to normalized window coordinates. We let

*W* be the width of the rendering window in pixel coordinates and

*H* be the height of the rendering window in pixel coordinates.

We then define the normalized error metric *PE_norm_*:

$$PE_{norm}\left(p\right)=\frac{dist2D\left(proj\left(p\right),r\left(p\right)\right)}{\sqrt{WH}}.$$ (2)

By construction, some of the point primitives will lie outside of the field of view and such points should generate no rendered pixels. Additionally, the error metric assumes that if a point does lie within the field of view, that there will be a single pixel rendered. So in addition to the error metric described above, we tabulate the number of primitives that fall into each of these categories:

**true-visible** – the point lies in the field of view and is rendered as a single pixel
**true-invisible** – the point does not lie in the field of view and no pixels are rendered
**false-visible** – the point does not lie in the field of view but it is rendered by a single pixel
**false-invisible** – the point lies in the field of view but no pixels are rendered
**multiple-visible** – multiple pixels are rendered We only calculate the point rendering error metrics for the true-visible case.

### 3.5 Error Metrics for Line Segment Primitives

3D Line segment primitives are regarded as one-dimensional objects described by two 3D end points. The pixels rendered for each segment are regarded as zero-dimensional objects (points) in the 2D pixel coordinate system of the screen.

**Fig. 2:**
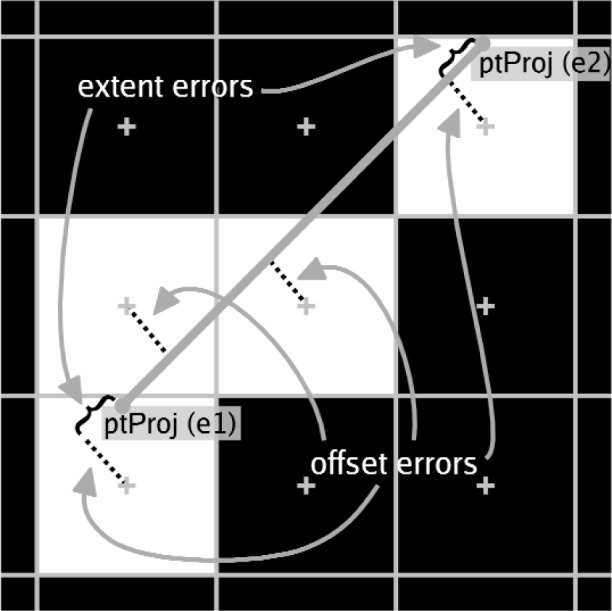
Components of the line segment error metrics. The white pixels are the pixels that were rendered to represent the line segment. The projection of the 3D line segment to 2D is shown as the gray line segment. The offset errors are the perpendicular distances from the centers of the rendered pixels to the projected line. The extent errors measure the difference between the ends of the projected line segment to the ends as indicated by the rendered pixels.

We devised error metrics for rendering of line segment primitives based on *offset errors* and *extent errors*. An offset error is the perpendicular distance from a rendered pixel to the 2D projection of the line defined by the two 3D segment end-points. These distances can be signed, where a distance from a 2D point to a 2D line is either positive or negative based on which side of the line the point lies. Extent errors measure how the set of rendered pixels cover the linear extent of the line segment. We identify two types of extent errors. When the rendered pixels extend beyond the end of the line segment, we refer to this as *overshoot*, when they do not cover to the end of the line segment, we refer to it as *undershoot*. Figure 2 illustrates the components of these metrics for a simple case.

Our error metric for line segments is implemented in a signed and unsigned form; we refer to these as *LSE_s_* and *LSE_u_* respectively. The signed metric (*LSE_s_*) uses signed offset errors and does not include extent errors. The unsigned metric (*LSE_u_*) regards all offset errors as positive and also includes extent errors. In order to specify these metrics we first define some terms. Let

**e1 and e2** be 3D points,
**(el, e2)** be the line segment with end points e1 to e2,
**ptProj (e)** be the 2D point produced by projecting the 3D point e to 2D pixel coordinates,
**lnProj (el, e2)** be the 2D line produced by projecting the 3D line determined by e1 and e2 to 2D pixel coordinates,
**P(e1, e2)** be the set of 2D pixels that are rendered for (el, e2),
**N(e1, e2)** be the number of pixels in P(e1, e2), and
**ds (p, line)** be the signed distance from 2D point p to a 2D line in pixel coordinates. This distance is positive when p is on one side of the line and negative when p is on the other side. When p is a pixel position, this distance is an offset error as described above.

As mentioned above, pixels are regarded as 2D points in this discussion. We now define the signed error metric LSE,:

$$LSE_{s}\left(e1,e2\right)=\left|\frac{\sum_{p\in P\left(e1,e2\right)}ds\left(p,lnProj\left(e1,e2\right)\right)}{N\left(e1,e2\right)}\right|.$$ (3)

This is the absolute value of the average of the offset errors for all rendered pixels, so *LSE_s_*, is in units of linear pixels. This measures the extent to which the offset errors are biased to one side or the other of the projected line segment. Note that by taking the absolute value of the average offset error, we are choosing to measure the magnitude of the bias without considering a direction to the bias.

In order to define *LSE_u_*, we must first define the extent errors: the amount that the set of rendered pixels overshoot or undershoot the projected line segment. We first observe that the set of rendered pixels, *P*(*e*1, *e*2), can be ordered based on the position of orthogonal projection of each pixel onto *lnProj*(*e*1, *e*2). We order these so that as we move from one pixel to the next, the projections of these pixels onto the line move in the direction from *ptProj*(*e*1) to *ptProj*(*e*2). In this ordering of the rendered pixels, the first in the list corresponds (in some sense) to e1, and the last in the list corresponds to e2. With this background, we can define the extent error. Augmenting the definitions above, let

**projToLn(p, 1)** be point on line 1 closest to point p,
**first** be the first point in the set P(e1,e2) when ordered as described above,
**last** be the last point in the set e1,e2) when ordered as described above, and
**dist2D(a, b)** be the positive distance between 2D points a and b in pixel coordinates.
Then we define the extent error as follows:

$$\begin{array}{l} extentError\left(e1,e2\right)=\\ |dist2D\left(projToLn\left(first,lnProj\left(e1,e2\right)\right),ptProj\left(e1\right)\right)|+\\ |dist2D\left(projToLn\left(last,lnProj\left(e1,e2\right)\right),ptProj\left(e2\right)\right)|. \end{array}$$ (4)

We can now define the unsigned line segment rendering error metric:

$$\begin{array}{l} LSE_{u}\left(e1,e2\right)=\\ \frac{\sum_{p\in P\left(e1,e2\right)}|ds\left(p,lnProj\left(e1,e2\right)\right)|+extentError\left(e1,e2\right)}{N\left(e1,e2\right)+2} \end{array}$$ (5)

This is the average magnitude of the offset and extent errors in pixel coordinates.

As for the point rendering metric, we define normalized versions of the line segment error metrics. Using *W* and *H* as defined in Sec. 3.4,

$$LSE_{u-norm}\left(e1,e2\right)=\frac{LSE_{u}\left(e1,e2\right)}{\sqrt{WH}},$$ (6)

and

$$LSE_{s-norm}\left(e1,e2\right)=\frac{LSE_{s}\left(e1,e2\right)}{\sqrt{WH}}.$$ (7)

These metrics are in linear normalized window coordinates. The unsigned metrics give assessments of overall deviation of the rendered pixels from the projected line segment. The signed metrics indicate if the deviation is biased toward one side of the projected line segment.

As described in Sec. 3.4, we categorize each line segment rendering case as true-visible, true-invisible, false-visible, and false-invisible. (No multiple-visible category is used for line segments.) We calculate our error metrics only on the true-visible cases.

### 3.6 Error Metrics for Triangle Primitives

3D triangle primitives are regarded as 2D objects described by three 3D vertex points. The pixels rendered for each triangle are regarded as 2D objects in the 2D pixel coordinate system of the rendering window. Each pixel is treated as covering a 2D square with edges of length 1 that is centered at integral pixel coordinates. So the pixels form a square tiling of the screen area. This models the display device in an idealized form that no real display achieves. Nevertheless, pixels on real displays do occupy 2D areas, even if these areas do not perfectly conform to the square tiling of the screen described above. Our approach relates (however imperfectly) these areas to the area of the projected triangle primitive. One could construct a triangle rendering error metric based on regarding the pixels as points rather than areas; we may do this in future work.

Our basic error metric for rendered triangles (*TE*) is based on determining how much of each square pixel area is inside the projection of the triangle to the 2D viewing area. Again, we begin with some definitions. Let

**T** be a triangle determined by three vertex points,
**proj (T)** be the projection of the triangle to the 2D pixel coordinate system and clipped to the viewing area,
**p** be a pixel,
**P(T)** be the set of pixels rendered for T,
**S** be a 2D polygon,
**inside (S, p)** be the area of the pixel p that lies inside the 2D polygon S, and
**outside (S, p)** be the area of the pixel p that lies outside the 2D polygon S.

Note that for a given S and p,

inside(S,p)+outside(S,p)=1. (8)

Then we define two forms of the rendering error metric TE, a signed version (*TE_s_*) and an unsigned version (*TE_u_*) as follows:

$$\begin{array}{l} TE_{s}\left(T\right)=\sum_{p\in P\left(T\right)}outside\left(proj\left(T\right),p\right)\\ \;-\sum_{p\notin P\left(T\right)}inside\left(proj\left(T\right),p\right), \end{array}$$ (9)

and

$$\begin{array}{l} TE_{u}\left(T\right)=\sum_{p\in P\left(T\right)}outside\left(proj\left(T\right),p\right)\\ \;+\sum_{p\notin P\left(T\right)}inside\left(proj\left(T\right),p\right). \end{array}$$ (10)

Both of these metrics measure area in pixel coordinates.

**Fig. 3:**
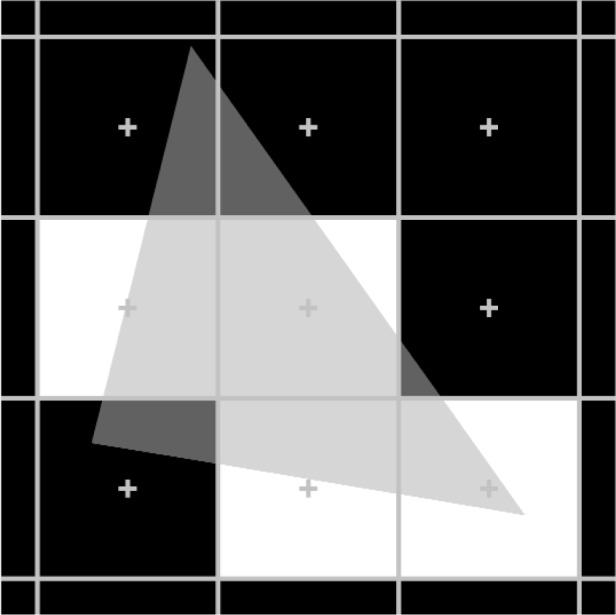
The components of the triangle error metrics. The white pixels are the pixels that were rendered for the 3D triangle. The projection of the 3D triangle is shown as an overlay. The areas of the white pixels that are outside the projected triangle and the areas of the black pixels that are inside the projected triangle represent errors.

Figure 3 illustrates the components of the triangle error metrics. In this figure, the projected triangle is shown overlaying the grid of pixels. The four light pixels are the pixels that were rendered to represent the 3D triangle. The light gray area represents the rendered area that correctly lies inside the projected triangle area and the black area is correctly outside the projected triangle. The white area corresponds to the first term of the two metrics above: it is rendered pixel area that is outside the projected triangle. The dark gray area corresponds to the second term: it is unrendered pixel area that is inside the projected triangle.

As for the line segment measures, the unsigned metric assesses the overall deviation of the rendered triangle from the projection of that triangle, while the signed metric indicates whether there is any bias in the errors. When *TE_s_* is positive, it means that errors are biased toward rendered pixels being outside the projected triangle. When *TE_s_* is negative, it means that errors are biased toward unrendered pixels inside the projected triangle.

We again specify normalized versions of these metrics in much the same way that we did for the line segment metrics. Because *TE_s_* and *TE_u_* are measures of area, we normalize based on the pixel area of the entire window yielding an area in normalized window coordinates. Letting *W* and *H* be defined as in Sec. 3.4, we define the normalized error metrics as follows:

$$TE_{u-norm}\left(T\right)=\frac{TE_{u}\left(T\right)}{WH},$$ (11)

and

$$TE_{s-norm}\left(T\right)=\frac{TE_{s}\left(T\right)}{WH}.$$ (12)

Another possible normalization of these metrics could be accomplished by dividing by the combined length (in pixel coordinates) of the visible edges of the triangle. This normalization is based on the hypothesis that triangle rendering errors are most likely to occur near the visible edges and thus would be proportional to the length of those edges.

Again, we use the categories true-visible, true-invisible, false-visible, and false-invisible. We calculate our error metrics only on the true-visible cases.

## 4. Results

We present results for each geometric primitive type, each associated error metric, and each platform. We use these error metrics to quantify and to summarize errors associated with rendering and as tools for comparing different rendering cases (different window sizes, platforms, and so on). We will present both the normalized and non-normalized metrics for the point primitive, but in order to simplify the presentation, we will focus on the normalized form of each metric for line segments and triangles. The same sorts of analyses, comparisons, and plots can be done with the non-normalized metrics. Indeed, in some contexts it might be more appropriate to look at the non-normalized metrics.

**Fig. 4:**
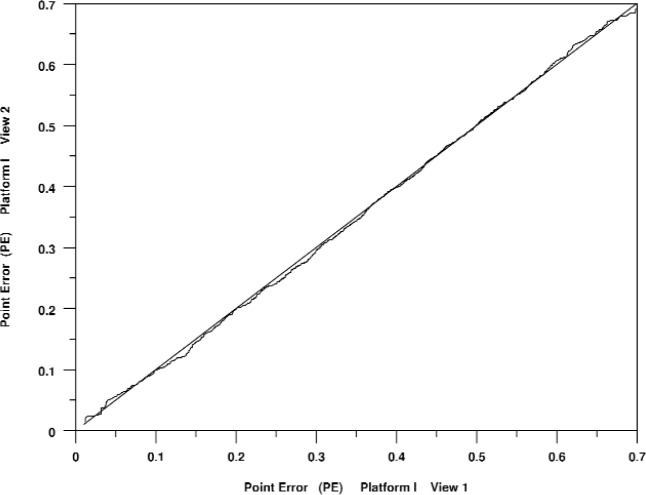
Quantile-quantile plot of point rendering errors measured for View 1 on Platform I versus View 2 on Platform I.

In analyzing the results of these tests we found that there was very little effect in changing the camera orientation. This means that although there were eight distinct sets of viewing parameters, the views that varied only by the camera orientation produced results that were, in aggregate, practically identical. Figure 4 illustrates this graphically with a quantile-quantile plot of point errors for for View 1 versus View 2 on Platform I. (Recall that Table 1 indicates that View 1 and View 2 differ only in camera orientation.) This example is fairly typical of comparisons of views that differ only by camera orientation.

We used the usual *t* tests and *F* tests to try to confirm that these pairs of camera orientation cases were equivalent in both mean and variance for each error metric that we present. Although the *t* test is robust in the presence of non-normal distributions, the *F* test is not, so we supplemented the *F* test with the Levene test of variance, which is more robust for non-normal data. We applied these tests both to the raw data as well as to Box-Cox transformed data when possible. We found that the *t* tests confirmed that the locations of the distributions for all of the pairs of camera orientation cases were equivalent. However for some cases, the *F* tests indicated a difference in variation for the raw data and/or the Box-Cox transformed data; the Levene tests, however, indicated a difference in only a single case. At the same time, we observed that for these cases, the differences in standard deviations were small compared to the magnitude of the standard deviations themselves. Thus, for practical purposes, we decided to regard cases that differed only in camera orientation as equivalent.

So, in the interest of brevity, we report results for four view groups, which we designate A, B, C, and D. View A is the aggregate of views 1 and 2; View B is the aggregate of views 3 and 4; View C is the aggregate of views 5 and 6; and View D is the aggregate of views 7 and 8. As mentioned above, we tested 100,000 of each primitive type for each of the eight original views, so after aggregation, we have run 200,000 of each primitive type for each of the aggregated views on each platform.

When summarizing the results, we will present the median and the interquartile range (IQR) rather than the mean and standard deviation. This is done because the distributions of these measurements are not normal and some of them contain a small number of extreme outliers. These outliers tend to have undue influence on the mean and standard deviation while having little effect on the median and IQR. We will present histograms to illustrate and to compare the shapes of the distributions.

### 4.1 Results for Point Primitives

First, we present the visibility counts for each of the aggregated views in Table 2.

There is a very close correspondence between the two platforms. We note that views A and C have angular fields of view of 30 degress, while views B and D have angular fields of view of 150 degrees. We expect a larger number of points to be visible given a wider field of view.

We see that an extremely small number of points are visibly rendered when they should be off-screen, while there is a low level (between 0.005 % and 0.08 %) of points that are invisible when they should be rendered.

Now we present the statistics on the errors (*PE*) found for each of the points in the true-visible category. Table 3 shows the statistics for the point rendering error metric *PE* for each view and platform. Table 4 shows statistics for the normalized form of the metric (*PE_norm_*).

**Fig. 5:**
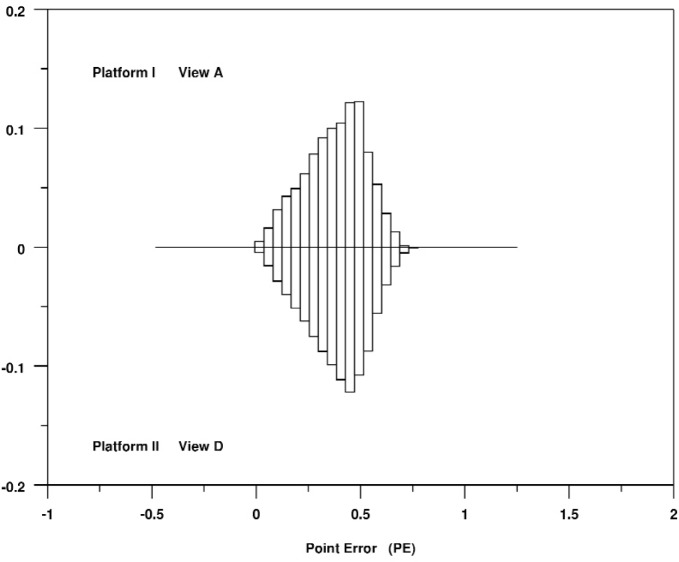
Relative bihistogram of the point rendering error metric *PE* as measured for View A on Platform I versus View D on Platform II.

First of all, we see a remarkable consistency among all of the views and platforms for *PE*. Not only are the centers and spreads of the distributions consistent across all cases, but the shapes of the distributions are very consistent. For example, Fig. 5 shows a relative bihistogram that compares the distribution of *PE* for View A on Platform I with View D on Platform II. We also note that this distribution is very close to the distribution that one would expect if the X and Y components of the rendering errors were uniformly distributed in the interval [−0.5, 0.5].

For the normalized metric *PE_norm_*, we see the results for *PE* scaled by the normalization factors based on window size. This, of course, produces errors that are smaller for the larger window size. This reflects the usual understanding that a higher resolution rendering is more accurate than a lower resolution rendering.

We note again that *PE* is expressed in pixel units while *PE_norm_* is expressed in normalized window coordinates. *PE_norm_* is useful for comparing results in windows of differing sizes, while *PE* is somewhat more easily converted to physical units (such as millimeters) on an actual screen. For example, one of our liquid crystal displays (LCD) has a pixel spacing of 0.255 mm. This means that the median point rendering error of about 0.396 pixel units (Table 3) corresponds to a median error of approximately 0.101 mm in physical units.

### 4.2 Results for Line Segment Primitives

Table 5 shows the counts for the various visibility categories for each platform and view. We see a very low level of false visible and false invisible cases and very few differences between the platforms.

#### 4.2.1 Normalized Signed Line Segment Rendering Errors

We use the normalized signed line segment metric *LSE_s_*_−_*_norm_* as a measure of the extent to which the rendered pixels are biased to one side of the projected line segment. This metric is used to compare the different cases across platforms and across cases within platforms.

**Fig. 6:**
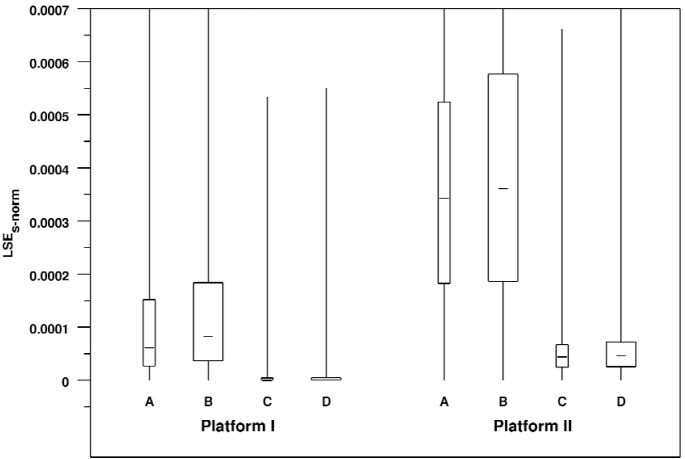
Box plot of normalized signed line segment rendering errors broken down by view and platform. Note that the range of the Y axis has been restricted so that the body of the box plots can be fairly compared. As a result, for some of distributions the maximum value lies outside of the plot area. The width of each box is proportional to the number of measurements in the corresponding distribution.

Table 6 shows the errors for each view for each platform. Figure 6 shows these errors graphically in the form of a box plot. We see that for both platforms, the 30 degree views have slightly smaller medians and spreads than the corresponding 150 degree views, although the effect is larger on Platform I. We also see that the smaller window size produces errors that have a larger median and spread than the larger window size; again, this effect is larger on Platform I.

**Fig. 7:**
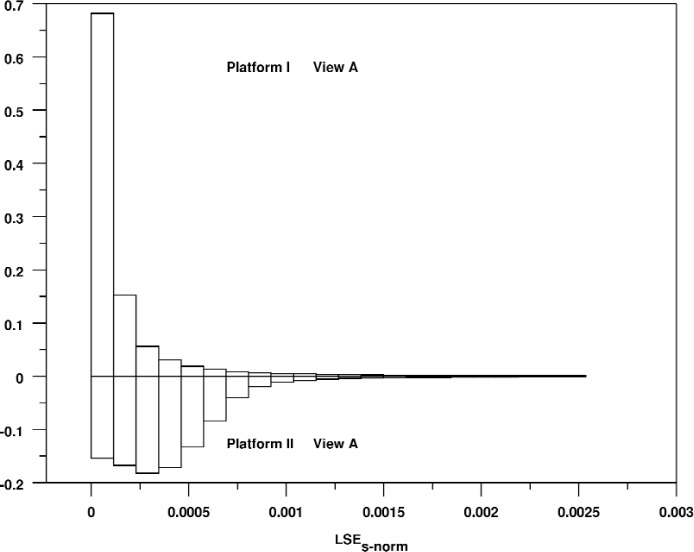
Relative bihistogram of the normalized signed line segment rendering error for View A and Platforms I and II.

It is also worthwhile to compare the shapes of these distributions. The shapes of the distributions are very consistent for the different views for a single platform, but they are quite different across platforms. For example, Fig. 7 shows a bihistogram of View A for the two platforms. The shape of these distributions is strikingly different. For Platform I, the distribution is closely clustered near zero with a rapid drop-off. For Platform II, the distribution stays fairly high and even shows a slight peak that is well away from zero.

To summarize, we see a higher level of bias in rendered line segments in the cases with smaller window sizes and we see a higher level of bias on Platform II than on Platform I for equivalent cases.

#### 4.2.2 Normalized Unsigned Line Segment Rendering Errors

**Fig. 8:**
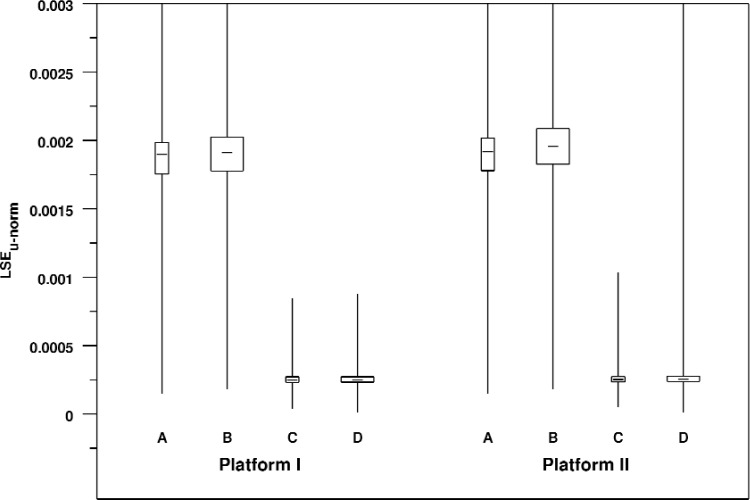
Box plot of normalized unsigned line segment rendering errors broken down by view and platform. As in Fig. 8, the range of the Y axis has been restricted so that the body of the box plots can be fairly compared. This results in some of the maximum values extending beyond the plot area.

We use the normalized unsigned line segment error *LSE_u_*_−_*_norm_* to assess the magnitude of the total error of the rendered line segment. It enables us to compare errors for the various views and platforms. Table 7 summarizes error statistics for each view and platform. Figure 8 shows the same data in graphic form.

**Fig. 9:**
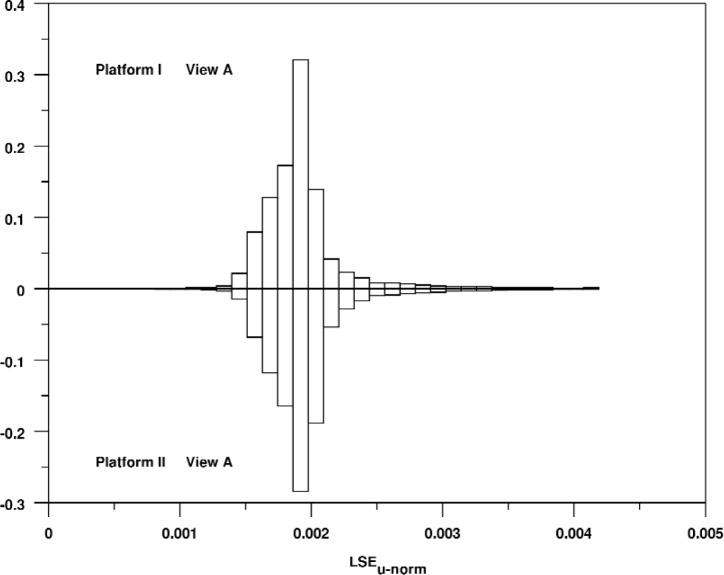
Relative bihistogram of the normalized unsigned line segment rendering error for View A and Platforms I and II.

The first thing that we see is that the two platforms give practically the same results for equivalent cases. Histograms of the various distributions confirm that the results from corresponding cases across platforms closely match. For example, Fig. 9 compares the histograms for View A on the two platforms. Furthermore we see that changing the angular field of view seems to produce no substantial difference. The major effect that we see is with a change in window size. The smaller window size produces a much larger measure of error with a much wider distribution than the larger window size.

Although there are, of course, large differences in normalized window coordinates between the two different window sizes, when we convert these measurements to physical units on a real display or to pixel coordinates the differences are very small. Using our LCD display with pixel spacing of 0.255 mm as an example, we see that all of the medians fall into the range between 0.059 mm and 0.065 mm. In somewhat broader strokes, we could also say that all of the median errors are a little less than a quarter of a pixel.

### 4.3 Results for Triangle Primitives

Table 8 shows the counts for the four visibility categories broken down by view and platform. The counts on Platform I closely track those on Platform II. As we expect, the number of visible triangles that are visible in the wider fields of view (Views B and D) is greater than counts for the narrower fields of view (Views A and C). We see no false visible cases and a relatively low level of false invisible cases, with the counts for the larger windows (Views C and D) being much lower than the smaller windows (Views A and B).

In the course of running these triangle rendering tests, we observe that sometimes a triangle covers the entire field of view. Typically, this results in every pixel in the window being rendered. We refer to this as a *full window rendering*. When this occurs, both the unsigned and signed error measures are exactly zero. Unsurprisingly, this occurs much more often for the 30 degree FOV cases than the 150 degree FOV cases. A full window rendering happens sufficiently often that it noticeably affects the distributions. We will point this out as it appears in the results that we present below.

#### 4.3.1 Normalized Signed Triangle Rendering Errors

**Fig. 10:**
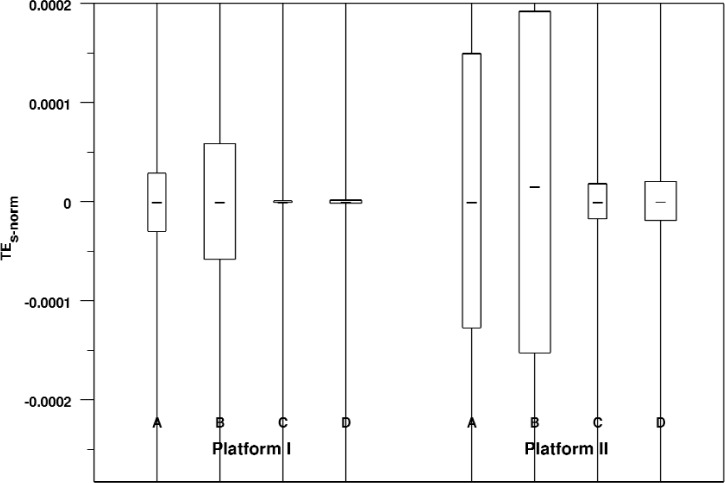
Box plot of normalized signed triangle rendering errors broken down by view and platform. Again, the range of the Y axis again has been restricted so that the body of the box plots can be fairly compared. This results in the maximum and minimum values lying outside of the plot area.

The normalized signed error metric *TE_s_*_−_*_norm_* measures the extent to which the rendered area is outside rather than inside the projection of the 3D triangle. A positive measure indicates that a greater error area is outside the projected triangle and a negative value indicates more error inside. Table 9 presents summary statistics broken down by view and platform. Figure 10 presents the same results in the form of a box plot. Note that the fact that the median for Views A and C is exactly zero is due to the substantial number of full window renderings that occur for the narrower field of view.

For all four views on both platforms the medians are all very close to zero. This indicates that there is little bias toward errors occurring outside versus inside the projected triangle. However there are differences between cases that show up when we look at the spread of the distributions. The spread of the distribution is substantially larger on Platform II compared to the same view on Platform I. Furthermore, the spread of the views for the larger window size is much less than for the smaller window size on both platforms; this is clearly related to the normalization.

**Fig. 11:**
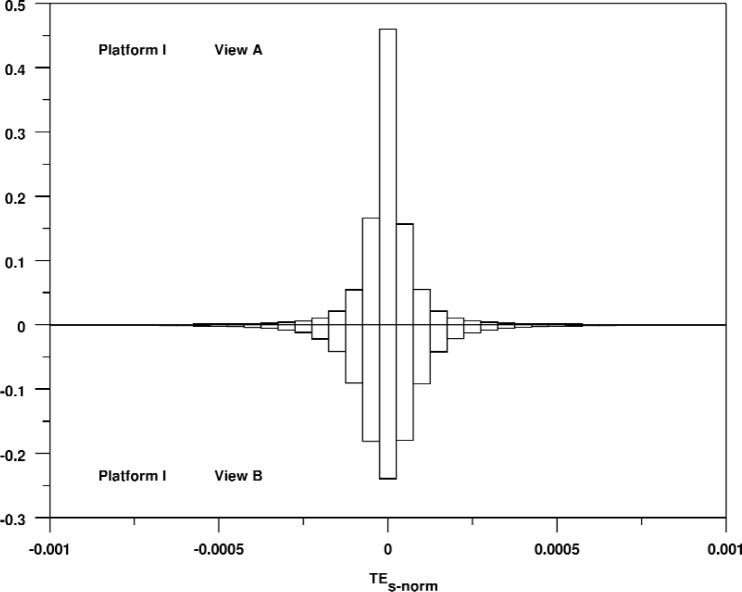
Relative bihistogram comparing the signed triangle rendering errors for Views A and B for Platform 1.

In Fig. 11, we show a relative bihistogram of these errors for Views A and B on Platform I. This shows the general shape of these error distributions, which is consistent across platforms and views. Note that while both histograms show a spike at zero, this peak is much more pronounced in the upper histogram, and the spread is somewhat narrower in the upper histogram. As mentioned above, full window renderings are much more common for views A and C than for views B and D. This results in a larger number of triangles with an error of exactly zero, which accounts for the more pronounced peak at zero for View A in the figure. This effect occurs on both platforms and both window sizes.

#### 4.3.2 Normalized Unsigned Triangle Rendering Errors

**Fig. 12:**
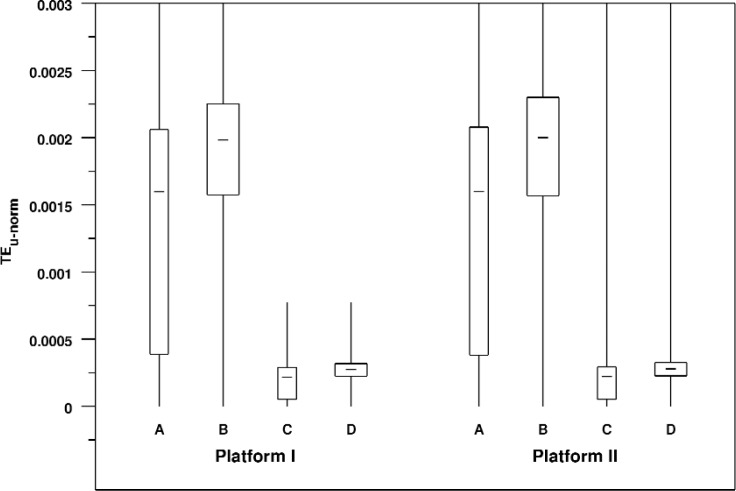
Box plot of normalized unsigned triangle rendering errors broken down by view and platform. As in previous figures, the range of the Y axis has been restricted so that the body of the box plots can be fairly compared. So for some of the distributions the maximum values lie outside of the plot area.

The normalized unsigned error *TE_u_* measures the total aggregate error area for the rendering. Table 10 summarizes these errors. Figure 12 shows the same data in the form of a box plot. We see that errors for the larger window size are much lower than those for the smaller window sizes, and errors for the wider FOV are slightly larger and more tightly grouped than for the narrower FOV. These effects are present for both platforms.

**Fig. 13:**
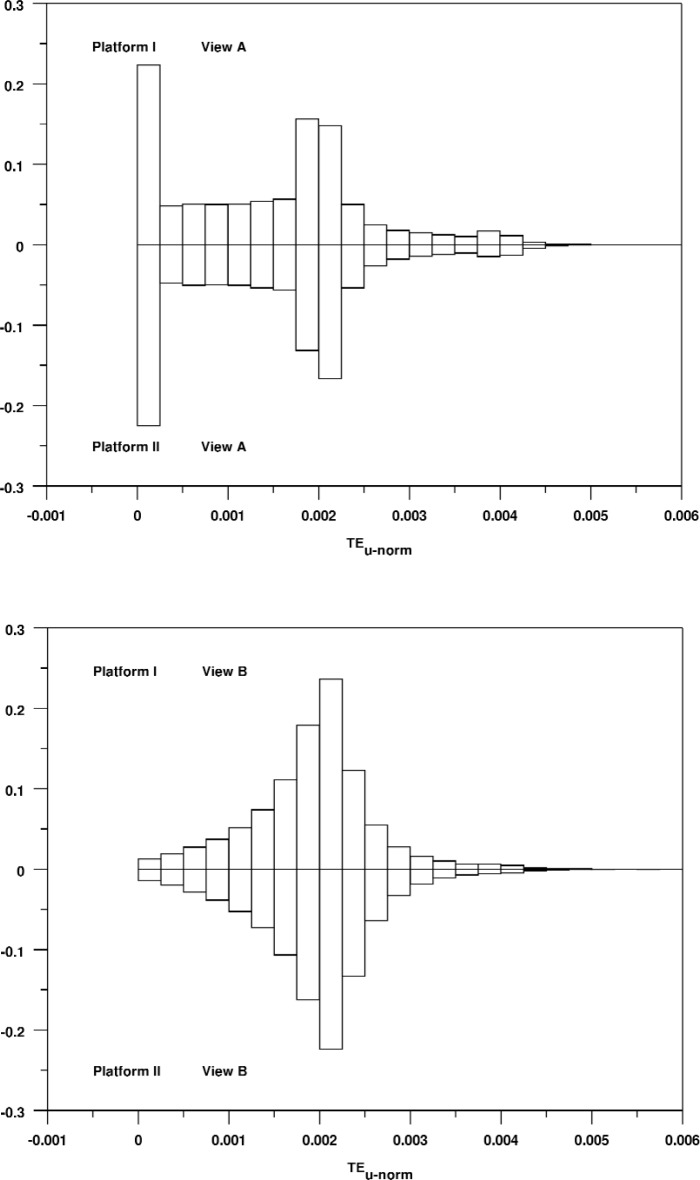
Relative bihistograms comparing the distribution of normalized unsigned triangle rendering errors for View A and B on Platform I versus Platform II.

In fact, we see a remarkable consistency between the two platforms in the appearance of these distributions. For example, Fig. 13 shows relative bihistograms comparing View A on Platform I versus Platform II and View B on Platform I versus Platform II. This figure shows both the equivalence of corresponding distributions across platforms and the characteristic shape of the distributions. Particularly interesting is the difference of the shapes of the distributions for Views A and B. View A and View B differ only in their angular fields of view (30 degrees versus 150 degrees respectively). The smaller field of view for View A results in many more full window renderings which accounts for the peak at zero, but it is unclear why the shape of the rest of the distribution is so different from that of View B.

To put these measurements into perspective, so to speak, we can convert the normalized errors into physical units. Because *TE_u_* is a measure of area, we can convert the medians from Table 10 into units of square millimeters (mm^2^) For example, the median of 1.606 × 10^−3^ in normalized window coordinates for View A on Platform I corresponds to an area of 1.71 mm^2^ on our LCD display with pixel spacing of 0.255 mm. Similarly, the median of 0.286 × 10^−3^ that we report for View D on Platform II corresponds to an area of 15.3 mm^2^. The same conversions can be applied to the IQRs as well as to the medians.

## 5. Conclusions and Future Work

The error metrics that we present above have enabled us to make quantitative estimates of errors in rendering the three most basic geometric primitives used in computer graphics. This lets us compare errors for different rendering cases, including across platforms. For example, we’ve seen that point rendering has a median error of about 0.39 in pixel coordinates, that Platform I has substantially less bias error in rendering line segments (*LSE_s_*_−_*_norm_*) than Platform II, and so on. Our normalizations provide a way of quantifying our intuitive understanding that higher resolution renderings produce smaller errors.

These results can provide useful information in real-world viewing situations. These measurements are easily converted to physical dimensions, as we have illustrated in Sec. 4 for an actual LCD display device. Of course, different displays have different physical characteristics and will show different errors when those errors are expressed in physical units. For example, Table 11 shows how the point rendering error (*PE*) of 0.396 pixel units translates to physical dimensions on three representative screens that we use in our group at NIST: a flat-panel LCD monitor, a CRT, and a large rear-projected screen that is used in our interactive immersive visualization (virtual reality) system.

Note that the error metrics that we present here do not capture all of the possible types of rendering errors that could occur. For example, the line segment rendering error metrics do not measure whether there are gaps in the rendered set of pixels. Similarly, there is no direct measure of whether a triangle is rendered as a simply connected set of pixels.

We plan to continue this work by quantifying errors in other aspects of the process of making visual representations of information. For example, we will be analyzing how these rendering errors result in quantifiable errors in depth representation in stereo displays. We will also be investigating errors that result from the process of generating renderable 3D geometric primitives from underlying data representations (for example, CT scans). We refer to these as *modeling errors*. For our work in immersive visualization environments, we will be looking at the viewing errors caused by the position tracking systems used in virtual reality systems. Finally, we are developing methods for aggregating all of these types of errors to form an overall quantitative assessment of errors contributed by the visualization process. It is our goal that these errors can, in turn, be aggregated with error assessments of the underlying data being presented to provide an overall uncertainty and confidence interval.

We believe that these tools enable us to understand better the possible errors in our displays of scientific data and other quantitative information. This understanding is critical for making informed judgements based on these visual representations.

## Figures and Tables

**Table 1:** The eight sets of viewing parameters

	Camera orientation	Angular field of view (degree)	Window pixel dimensions
View 1	CO-1	30	128×128
View 2	CO-2	30	128×128
View 3	CO-1	150	128×128
View 4	CO-2	150	128×128
View 5	CO-1	30	907×907
View 6	CO-2	30	907×907
View 7	CO-1	150	907×907
View 8	CO-2	150	907×907

**Table 2:** The counts of point primitives rendered in each visibility category for each view and platform

Platform	View	True visible	True invisible	False visible	False invisible
I	A	4225	195703	0	72
I	B	76711	123134	1	154
I	C	4225	195764	0	11
I	D	76711	123260	0	29
II	A	4224	195703	0	73
II	B	76704	123128	7	161
II	C	4225	195764	0	11
II	D	76710	123259	1	30

**Table 3:** Summary of non-normalized point rendering errors

View	FOV	Win Size	Platform I		Platform II	
			Median	IQR	Median	IQR
A	30	128	0.39555	0.20938	0.39553	0.21221
B	150	128	0.39716	0.20673	0.39714	0.20942
C	30	907	0.39542	0.20975	0.39542	0.21335
D	150	907	0.39946	0.20597	0.39945	0.20904

**Table 4:** Summary of normalized point rendering errors (×10^3^)

View	FOV	Win Size	Platform I		Platform II	
			Median	IQR	Median	IQR
A	30	128	3.090	1.636	3.090	1.658
B	150	128	3.103	1.615	3.103	1.636
C	30	907	0.436	0.231	0.436	0.235
D	150	907	0.440	0.227	0.440	0.230

**Table 5:** The counts of line segments in each visibility category for each platform and view

Platform	View	True visible	True invisible	False visible	False invisible
I	A	20540	179380	0	80
I	B	125260	74556	0	184
I	C	20608	179380	0	12
I	D	125424	74556	0	20
II	A	20538	179378	2	82
II	B	125249	74554	2	195
II	C	20603	179379	1	17
II	D	125424	74556	0	20

**Table 6:** Summary of normalized signed line segment rendering errors (×10^6^)

View	FOV	Win Size	Platform I		Platform II	
			Median	IQR	Median	IQR
A	30	128	62.71	125.63	344.89	341.96
B	150	128	84.55	146.92	362.60	390.40
C	30	907	1.67	3.47	45.39	42.99
D	150	907	2.24	4.00	48.05	46.60

**Table 7:** Summary of normalized unsigned line segment rendering errors (×10^3^)

View	FOV	Win Size	Platform I		Platform II	
			Median	IQR	Median	IQR
A	30	128	1.906	0.230	1.926	0.239
B	150	128	1.920	0.249	1.964	0.262
C	30	907	0.257	0.039	0.260	0.039
D	150	907	0.258	0.039	0.262	0.037

**Table 8:** The counts of triangle primitives rendered in each visibility category for each view and platform

Platform	View	True visible	True invisible	False visible	False invisible
I	A	52580	147133	0	287
I	B	155090	44236	0	674
I	C	52828	147133	0	39
I	D	155691	44236	0	73
II	A	52585	147133	0	282
II	B	155110	44236	0	654
II	C	52830	147133	0	37
II	D	155688	44236	0	76

**Table 9:** Summary of normalized signed triangle rendering errors (×10^6^)

View	FOV	Win Size	Platform I		Platform II	
			Median	IQR	Median	IQR
A	30	128	0.00	58.75	0.00	276.66
B	150	128	−0.05	116.67	15.48	344.57
C	30	907	0.00	1.58	0.00	35.45
D	150	907	−0.01	3.19	0.32	39.64

**Table 10:** Summary of normalized unsigned triangle rendering errors (×10^3^)

View	FOV	Win Size	Platform I		Platform II	
			Median	IQR	Median	IQR
A	30	128	1.606	1.673	1.607	1.698
B	150	128	1.989	0.680	2.007	0.732
C	30	907	0.226	0.237	0.229	0.241
D	150	907	0.281	0.093	0.286	0.099

**Table 11:** Point rendering error of 0.396 pixel units converted to physical units for three representative screens used at NIST

Display	PE
LCD	0.101 mm
CRT	0.118 mm
Projection	0.915 mm

## Footnotes

1 Certain commercial equipment, instruments, or materials are identified in this paper to foster understanding. Such identification does not imply recommendation or endorsement by the National Institute of Standards and Technology, nor does it imply that the materials or equipment identified are necessarily the best available for the purpose.
